# Sodium and Potassium Intake in Residents of Retirement Homes

**DOI:** 10.3390/nu12092725

**Published:** 2020-09-06

**Authors:** Boštjan Rejec, Petra Golja, Cirila Hlastan Ribič, Matjaž Klemenc

**Affiliations:** 1 MG d.o.o., Ulica Nikole Tesle 3, 5290 Šempeter pri Gorici, Slovenia; 2 Department of Biology, Chair of Physiology, Anthropology and Ethology, Biotechnical Faculty, University of Ljubljana, Večna pot 111, 1000 Ljubljana, Slovenia; petra.golja@bf.uni-lj.si; 3 National Institute of Public Health, Trubarjeva 2, 1000 Ljubljana, Slovenia; 4 Intensive Care Unit, General Hospital Nova Gorica, Ulica padlih borcev 13A, 5290 Šempeter pri Gorici, Slovenia; klemenc.matjaz@gmail.com

**Keywords:** sodium, salt, potassium, intake, residents, retirement homes

## Abstract

Excessive salt intake and its impact on health is a public health problem in many regions of the world. The currently estimated dietary intake of salt among free-living adults is well above the WHO recommendations. Over the years, the number of residents in retirement homes has increased. Besides this, the nutrition of elderly people may be affected by physiological changes that occur with aging. The question is whether residents of retirement homes receive a more balanced diet, or whether the trend of excessive salt consumption continues even among institutionalised elderly people. Salt and potassium intake were assessed by measuring sodium and potassium excretion over 24 h in urine collected from a sample of residents of three retirement homes in the Goriška region, Slovenia. The average salt intake was 8.3 (2.9) g/day, which was significantly higher (*p* < 0.001) in men than in women (10.1 (3.1) vs. 7.3 (2.2) g/day, respectively). The estimated total daily potassium intake was 2.6 (0.6) g/day in men and 2.0 (0.8) g/day in women (mean 2.2 (0.8) g/day). The ratio of sodium to potassium was 1.53 (0.48). The salt intake among residents of retirement homes in the Goriška region, especially in men, exceeds the WHO recommended daily intake of <5 g. The mean daily potassium intake was below the WHO recommendations of 3.5 g/day.

## 1. Introduction

Excessive salt (NaCl) intake and its impact on health is a public health problem in many regions of the world [1,2,3,4]. The currently estimated dietary intake of salt is about 9–12 g per day in most countries [1], which is well above the WHO recommend intake of less than 5 g of salt per day [2].

A high salt intake is the major cause of raised blood pressure, thereby increasing the risk of cardiovascular disease (CVD) [3,5,6,7,8]. Dietary salt intake is also associated with other diseases like gastric cancer [9,10,11,12] and obesity [13,14,15].

Salt is involved in nervous and muscular function, and in autoregulation of the body water balance [16]. Furthermore, sodium is an essential mineral in humans and, according to the national reference values for nutrient intake, its adequate daily intake for adults is 1.5 g (i.e., 65 mmol) [17]. Additionally, salt is a flavouring agent and acts as a food preservative both in industrial food processing and home cooking [18].

In Slovenia, the joint WHO/FAO recommendations for salt intake have been adopted, which state that the daliy individual intake of salt should be less than 5 g/day (or <2 g Na/day), while ensuring that the ingested salt is iodised [19]. Nevertheless, in 2007, the average salt intake in Slovene adults (aged 25–65 years) was 11.3 g/day, and was higher (*p* < 0.001) in men than in women (13.0 (5.1) vs. 9.9 (4.3) g/day, respectively) [20].

In contrast, a higher dietary potassium intake is associated with a lower blood pressure and a lower risk of death and cardiovascular events [6,8,21,22]. Potassium is also an essential nutrient. It is the most abundant cation in intracellular fluid, where it plays a key role in maintaining cell function, particularly in excitable cells such as muscles and nerves [23,24]. Furthermore, it is considered to be a marker of a healthy diet, since it is a nutrient found in fruits and vegetables [25]. The WHO suggests a potassium intake of at least 90 mmol/day (i.e., 3.51 g/day) for adults [26], while the Slovenian National Institute of Public Health recommends a daily intake of 4 g for adults and the elderly [17].

Emerging evidence suggests that an increased dietary sodium-to-potassium ratio (Na/K) is more strongly associated with an increased risk of hypertension and CVD than Na and K considered separately [27,28,29]. There is no generally accepted recommended guideline for the Na/K ratio. However, if the WHO guidelines on the recommended sodium and potassium intake are followed, the molar ratio of sodium to potassium (87 mmol/day: 90 mmol/day, for Na and K, respectively) would be approximately one to one [2,26].

Demographic projections suggest that in 2060 almost every third Slovenian resident will be 65 years old or over. Due to the aging population in Slovenia, the number of residents in retirement homes has been increasing. Over the past eight years, the number of residents in retirement homes has increased by 15% [30]. In retirement homes, residents are supposed to receive regular and nutritionally appropriate meals.

Physiological changes that occur with aging may affect the nutrition of elderly people [31,32,33]. Various chronic diseases may affect appetites and the ability to eat certain foods. The side effects of certain drugs, such as anorexia, nausea, altered flavor, and food–drug interactions, may also affect feeding and cause malnutrition [33]. This may all be reflected in the salt and potassium intake. All these circumstances raise the question of whether residents of retirement homes receive a more balanced diet compared to the noninstitutionalised population, or whether the trend of excessive salt consumption continues even among institutionalized elderly people, despite meals being prepared by professional cooks.

There are several methods for evaluating the salt and potassium intake of an individual, including dietary recalls (or dietary records) from the last 24 to 96 h, a food frequency questionnaire (FFQ), and 24 h urine collection aimed to evaluate sodium excretion. The latter is considered to be the most reliable method and is considered the "gold standard" for the estimation of salt and potassium intake [34,35,36,37,38,39,40].

While most studies have focused on salt and potassium intake among noninstitutionalised adults and children, our research was carried out among inscoptitutionalized people 59–100 years old. We sought to measure sodium excretion as a marker for salt intake, as well as potassium excretion as a marker for potassium intake in urine samples, collected over 24 h, of residents of retirement homes in the Goriška region, Slovenia. We also investigated salt intake relative to residence, age, and body mass index (BMI). We hypothesised that residents of retirement homes do not meet the WHO recommendations for salt and potassium intake. As their food is prepared by professional cooks, we also hypothesised that they consume less salt than the noninstitutionalised Slovene adult population. Finally, we hypothesised that they have a better Na/K ratio than the noninstitutionalised population due to regular daily consumption of freshly prepared food and limited intake of processed food high in sodium.

## 2. Materials and Methods

### 2.1. Participants

The participants were residents of three retirement homes in the Goriška region of the western part of Slovenia. Residents with diseases such as Addison’s disease, Cushing’s syndrome, diabetes insipidus, renal failure [41] and those who were taking medicines that could significantly affect sodium balance (such as vasopressin receptor antagonists, chlorthalidone, vasopressin, etc.) [41,42,43] were excluded from participation in the study. Residents with urinary incontinence were also excluded, except for those with a permanent urinary catheter.

The participants received written invitations to take part in the study, including an explanation of the purpose and protocol of the study, a request not to change their diet immediately before or during the study, and a consent form. The invitation also included information on the prevalence of chronic diseases in Slovenia and worldwide, particularly information on high salt intake as a risk factor. Participants who signed and returned the consent forms received a letter of appreciation and detailed instructions about the 24 h urine collection procedure [44].

Urine samples were collected in December 2017, March 2018, and November 2018. The study protocol was approved by the Medical Ethics Committee of the Republic of Slovenia (No. 0120-586/2017/4).

### 2.2. Anthropometric Measurements

On the day before urine collection, each participant’s body mass and height was measured with a medical grade scale and stadiometer (Seca 767, Hamburg, Germany) and participants’ ages were noted.

### 2.3. Urine Collection (24 h) and Measurements of the Urine Volume, Sodium, Potassium and Creatinine Excretions

To determine daily sodium and potassium excretion, one 24 h urine collection per participant was used. The urine volume and creatinine excretion were determined. All procedures were conducted in accordance with the existing guidelines [44].

The urine collection was performed by collecting participant’s urine in a special container over a 24 h period. On the day of the test, the participants were asked to void the bladder in the morning, discard the urine and note the time as the beginning of the 24 h urine collection period. All urine voided in the next 24 h was collected in the same container. The collected urine samples were transported to the Department of Laboratory Diagnostics, General Hospital Nova Gorica, Slovenia, the same morning as the end of the 24 h urine collection period.

Creatinine excretion in urine occurs at a fairly constant rate over 24 h. A typical 70-kg adult man produces about 2 g of creatinine per day and there is a continual production and excretion of creatinine in the urine [45]. The 24 h creatinine excretion in urine was therefore used as a measure for excluding any urine collection samples judged to be incomplete according to creatinine values [46,47,48,49,50]. Creatinine was measured using the Jaffe method [51]. Thus, a 24 h urine collection was accepted for further analysis, if the creatinine excretion was 10.0–17.0 mmol/day for men and 7.5–12.0 mmol/day for women aged 45–60 years, or 8.5–14.25 mmol/day for men and 5.75–10.5 mmol/day for women aged 60–75 years, or 4.5–11.0 mmol/day for men and 3.5–8.5 mmol/day for women aged over 75 years [52]. Furthermore, urine collections were not considered appropriate and were thus not analysed if the 24-h urinary volumes were smaller than 250 mL [53].

The sodium and potassium 24 h excretions were determined by indirect potentiometry [54], while considering the total volume of urine collected (litres; L) and the precise time of urine collection. The sodium and potassium excretions were calculated individually as the product of the urine sodium or potassium concentration (mmol/L) and 24 h urinary volume (L/day), and were expressed in mmol/day. The daily Na and K excretion estimates (g/day) were calculated by multiplying the urine sodium or potassium concentration (mmol/day) with the respective atomic weights (Na: 23 mg/mmol and K: 39.1 mg/mmol) [55].

Considering that about 90% of the ingested sodium [37,56,57] and potassium [23,24] is excreted in urine and the remaining 10% is excreted in faeces and sweat, the total daily intakes of sodium and potassium were callculated by dividing the daily Na and K excretion estimates (g/day) by 0.9.

For the conversion from sodium (Na) to sodium chloride (NaCl, i.e., salt), a factor of 2.54 was used (NaCl (g) = Na (g) × 2.54) [58].

### 2.4. Statistical Analyses

The analyses included descriptive and inferential statistical methods. The main group comparisons were performed using an χ^2^ test. To obtain information about the relationships between variables (salt intake, BMI, age), a Pearson correlation coefficient was calculated. After a preliminary F-test of homogeneity of variances was completed, a Student *t*-test was used to compare the mean values of variables between different groups (men vs. women). All significance levels presented in the present study are two-sided. The level of 0.05 was adopted as statistically significant.

## 3. Results

### 3.1. Sample Data (N)

**Figure 1:**
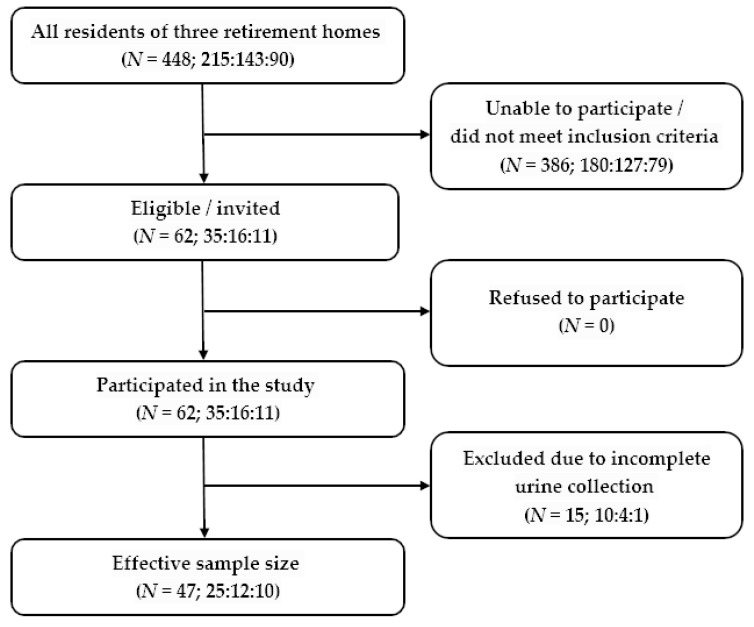
Study flow chart—62 residents of retirement homes were recruited to participate in the study, as they met the inclusion criteria. The effective sample size after the exclusion of subjects with incomplete urine collection was 47 participants.

The study included residents of three different retirement homes, who were able to participate in the study and met the inclusion criteria—62 in total. A total of 15 urine samples were later excluded from the analysis due to incomplete urine collection, as determined by creatinine measurements. The effective sample size for the determination of sodium and potassium excretion in 24 h urine samples was thus 47 participants (Figure 1).

The age, anthropometric characteristics and urine analysis results of the 47 residents of retirement homes are presented in Table 1.

### 3.2. Urine Volume and Creatinine Excretion

**Figure 2:**
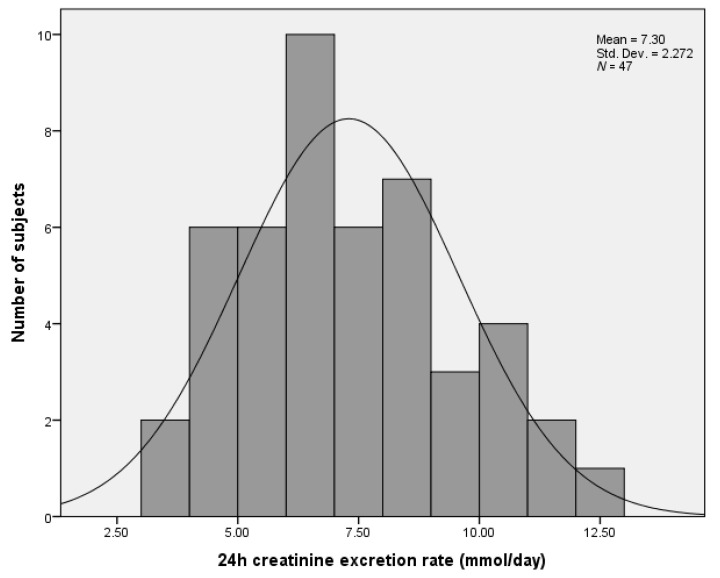
Number of retirement home residents relative to their 24 h urinary creatinine excretion values.

Participants’ average (SD) urine volume was 1.51 (0.48) L/day. Men’s urine volume was significantly higher (1.75 (0.38) L/day) than women’s (1.37 (0.48) L/day; *p* = 0.008, Table 1). Urinary creatinine excretion was also significantly higher in men (9.4 (1.8) mmol/day) than in women (6.1 (1.5) mmol/day; *p* < 0.001, Table 1). After the exclusion of incomplete urine samples, the coefficient of variation for urinary creatinine excretion, calculated across both genders and all age groups, was 31.3%. Figure 2 demonstrates the distribution of the 24 h urinary creatinine excretion values.

### 3.3. Sodium and Potassium Excretion in the Residents of Retirement Homes in the Goriška Region, Slovenia

**Figure 3:**
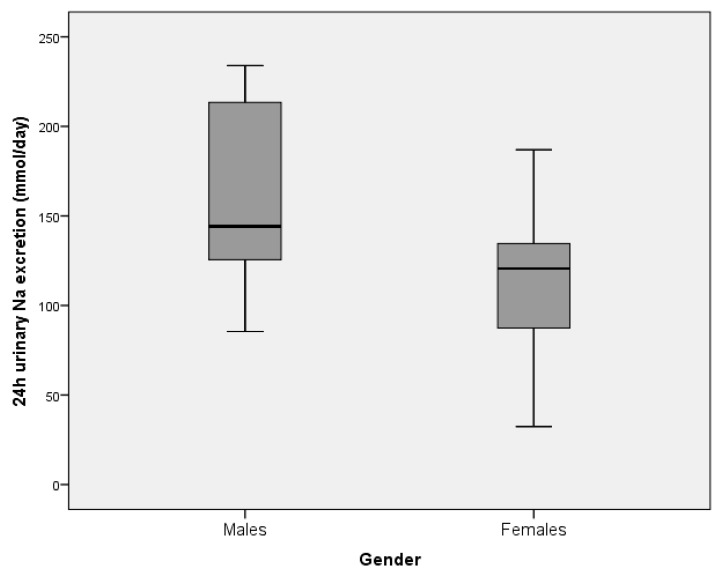
The estimate of mean urinary sodium excretion (over 24 h) in residents of retirement homes in the Goriška region, presented by gender (males: *N* = 17, females: *N* = 30). Boxes indicate medians (line inside box) and quartiles (upper and lower margins of the boxes); 95% CI are represented by vertical lines.

Urinary sodium excretion was significantly higher in men (155.3 (47.8) mmol/day) than in women (111.8 (34.1) mmol/d; *p* < 0.001, Figure 3). The estimated total daily salt (NaCl) intake was 10.1 g/day in men and 7.3 g/day in women (mean 8.3 g/day). Overall, according to the frequency distribution of the estimated daily salt intake data, 89.4% of the participants had a daily intake higher than the recommended 5 g/day (100% of men, 83.3% of women).

Moreover, urinary potassium excretion was significantly higher in men (60.5 (13.6) mmol/day) than in women (46.1 (17.7) mmol/day; *p* = 0.006). The estimated total daily potassium intake was 2.6 (0.6) g/day in men and 2.0 (0.8) g/day in women (mean 2.2 (0.8) g/day). The ratio of sodium to potassium was 1.53, with no significant difference between genders (1.54 (0.46) vs. 1.52 (0.50); *p* = 0.886).

The average salt intakes between residents of the three retirement homes were not significantly different (8.8 (2.9) g/day vs. 7.0 (1.4) g/day vs. 8.3 (3.6) g/day; *p* = 0.263).

### 3.4. Salt Intake and Potassium Excretion Relative to Age and BMI

**Figure 4:**
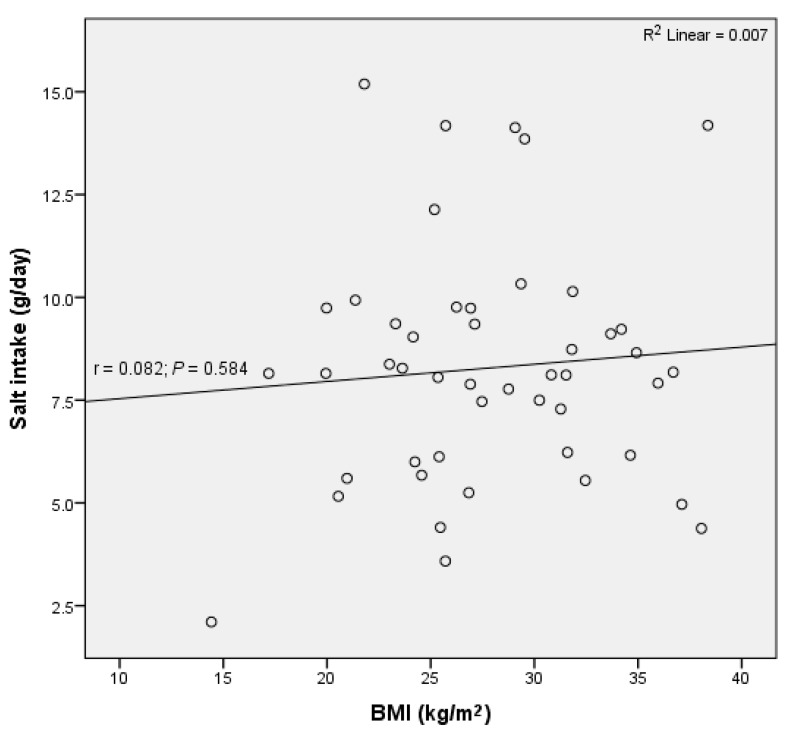
No significant correlation between daily salt intake and body mass index (BMI) was observed in residents of retirement homes in the Goriška region.

There was no significant correlation between BMI and daily salt intake or potassium excretion (R^2^ = 0.007, *p* = 0.584; R^2^ = 0.05, *p* = 0.132, respectively) (Figure 4).

**Figure 5:**
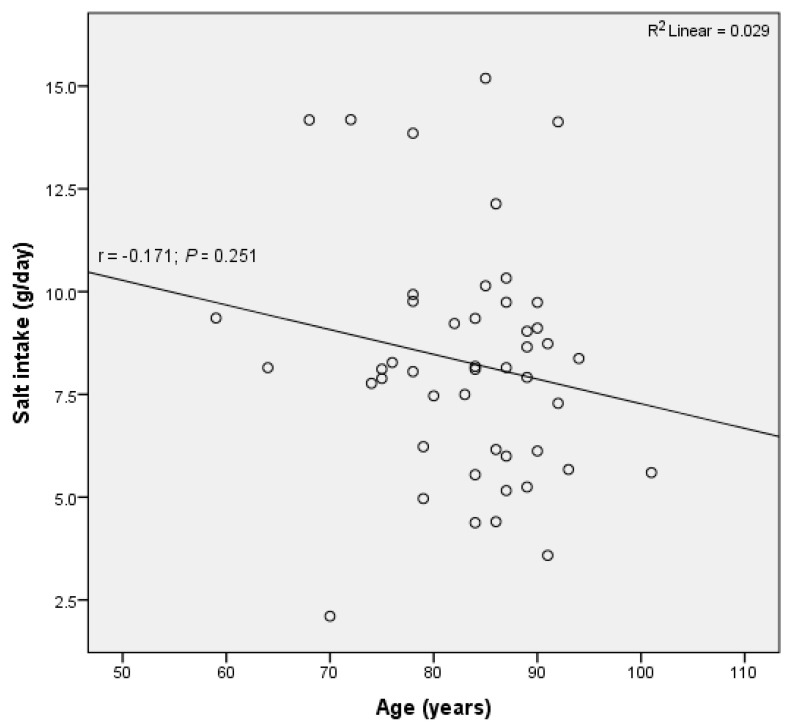
No significant correlation between daily salt intake and age was observed in residents of retirement homes in Goriška region.

Similarly, there was no significant correlation between age and salt intake (R^2^ = 0.029, *p* = 0.251) (Figure 5), nor between age and potassium excretion (R^2^ = 0.027, *p* = 0.268).

## 4. Discussion

The present study evaluated urinary sodium and potassium excretion over 24 h to provide estimates of the salt intake among residents of retirement homes in the Goriška region, Slovenia. Residents of retirement homes did not meet the WHO recommendations for salt and potassium intake, but they had a better Na/K ratio than the noninstitutionalised population.

The 24 h urinary sodium excretion in the Slovenian national salt intake survey, performed in 2007 among adults aged 25–65 years, was significantly higher than in our population (191.6 mmol/day vs. 127.6 mmol/day; *p* < 0.001), while a significant difference between genders was also observed in that survey (220.9 mmol Na/day in men and 169.8 mmol Na/day in women) [20].

The INTERSALT study, performed in 52 countries around the world, collected data on 24 h urinary sodium excretion in 10,079 men and women aged 20–59 [27]. It demonstrated that more than 50% of men had an average intake of sodium ranging from 150 to 199 mmol/day, and approximately 50% of women from 100 to 149 mmol/day [60]. The mean 24 h urinary Na excretion in the INTERSALT study was 156.0 mmol/day [61]. Values over 200 mol/day in men were observed in Canada, Colombia, Hungary, Ladakh (India), Bassiano (Italy), Poland, Portugal, and the Republic of Korea [60]. In a nationally representative cross-sectional study from 2014, on a sample of US adults aged 20–69, the estimated mean sodium excretion was found to be 3.608 g/day: 4.205 g/day in men and 3.039 g/day in women [62], which suggests a mean daily intake of sodium of 156.9 mmol/day.

Sodium excretion in our study population was lower than in the Slovene adult population [20], as well as in the majority of countries included in the INTERSALT studies [27,60,61] and the US [62]. This supports our hypothesis that institutionalised adults consume less salt than noninstitutionalised adults. The food offered in retirement homes is freshly prepared by professional cooks and therefore residents consume only little amounts of processed food (if any), which is high in sodium, which is a likely cause of the observed results. The difference in sodium excretion between genders, reported previously and also observed in our study, most likely reflects the differences in energy needs [17,63] and energy intake between men and women [57,64]. On average, men have higher energy needs and energy intake than women, therefore it is reasonable to assume that they also consume more sodium.

The estimated total daily salt intake of the participants of the present study exceeded the WHO recommended population salt daily intake of less than 5 g [2]. The average salt intake was not significantly different between residents of different retirement homes.

Similar to the results of the present study, the average salt intake in the Slovene adult population was 11.3 g/day, with a significantly higher intake also observed in men than in women (13.0 vs. 9.9 g/day, respectively) [20]. In neighboring countries, the mean salt intake among adults was 11.2 g/day for men and 9.6 g/day for women in Hungary [4], while in Italy the mean salt intake was 10.9 g/day for men and 8.5 g/day for women [65]. The salt intake in our study was more similar to the salt intake of Italian adults than that of Hungarian adults.

In the INTERSALT study, the mean 24 h urinary potassium excretion was 55.2 (25.3) mmol/day: 60.4 (27.5) mmol/day in men and 49.9 (21.7) mmol/day in women [61]. In the US, the estimated mean potassium excretion was 55 mmol (i.e., 2.2 g) per day in adults [62]. Among Italians aged 35–79, the mean 24 h potassium excretion was 63 mmol (i.e., 2.5 g) for men and 55 mmol (2.2 g) for women [65]. The mean urinary potassium excretion in the present study was lower than in the studies listed above, probably due to a lower food intake. Namely, the daily food intake decreases with aging [66,67] and therefore residents of retirement homes are likely to consume less potassium than the younger population.

In our study, potassium intake was below the Slovenian National Institute of Public Health recommendation for potassium intake, which is stated at 4 g/day, and below the WHO recommendation of at least 90 mmol potassium per day (i.e., 3.51 g/day) [17,26]. We assume that the observed difference in potassium intake between genders depends on the differences in energy needs [17,63] and energy intake between men and women [57,64].

In the INTERSALT study, the mean 24 h urinary Na/K ratio was 3.29 for men and 3.20 for women [61]. Among US adults aged 20–69, the mean sodium/potassium ratio was 3.17 [62]. The mean urinary sodium/potassium ratio among Italians aged 35–79 was 3.2 for men and 2.8 for women [65]. In our study, the Na/K ratio was favourably lower than in other studies, suggesting that our institutionalised participants had a more balanced diet—that is, they consumed food with more potassium and less sodium, as compared to the noninstitutionalised participants of the other studies.

The effect of aging on salt intake remains to be resolved. The majority of studies on this topic report increased taste detection thresholds in the elderly [68], which is particularly true for salty taste [69,70]. This may, in turn, promote the consumption of salt [71]. The use of total dental prosthesis, which is common in the elderly, can also diminish taste perception [72] and may thus promote salt intake [73]. In contrast, a diminished taste perception may reduce the interest in food and consequently decrease the overall food (and salt) intake [74]. Indeed, a diminished food intake with aging has already been reported [66,67]. In US adults aged 20–69, 24 h urinary sodium excretion did not differ significantly with age when subjects were grouped into two age groups (20–44 and 45–69 years) [62]. Yet, the authors reported that in a subsequent exploratory analysis 24 h urinary sodium excretion “appeared to be” lower in the oldest age group (60–69 years of age) [62], but provided no statistics. Similarly, in Italians aged 35–79, no differences in 24 h urinary sodium excretion were observed with age in either men or women, although the authors reported a trend towards an increased potassium excretion with age [65]. In the present study, no significant correlation between participants’ salt intake or potassium excretion and age was observed. Similarly, no significant correlation between participants’ age and salt intake was observed in the Slovenian national salt intake survey on adults aged 25–65 years [20]. It may therefore be reasonable to assume that age per se is not one of the most important factors determining salt intake, although factors such as a decreased taste perception or wearing total dental prosthesis may play a role.

Several hypotheses have been proposed to link salt consumption and obesity [13,14,15]. Namely, salt intake has been associated with an increased risk of obesity through increased sugar-sweetened beverage consumption in children and adolescents [3,15], as a higher sodium intake drives a thirst response and promotes fluid intake [75,76]. As a considerable proportion of fluid intake takes place in the form of soft drinks, an increase in soft drink consumption has been associated with an increase in body mass index [3]. Nevertheless, no significant correlation was observed between BMI and daily intake of salt or potassium excretion in the present study. Similarly, Oh (2017 [77]) observed that sodium excretion was not associated with obesity in elderly Koreans, while in the Slovenian adult population the intake of salt significantly increased with an increasing BMI [20]. We might speculate that elderly people exhibit a decreased thirst sensation [78] and consequently do not consume so many sugar-sweetened beverages than younger people. In our study, the urinary volume was significantly lower than in the Slovene adult population (1.51 L/d vs. 1.93 L/d; *p* < 0.0001) [20]. Considering that fluid intake is the primary determinant of urinary volume [79], it seems reasonable to assume that fluid intake among participants in the present study was lower than in the Slovene adult population. Furthermore, the food in retirement homes is freshly prepared by professional cooks and residents consume little processed food high in sodium, therefore fluid intake is likely less promoted by salty food than in the average adult Slovene population.

As we were studying not a general, but elderly institutionalised population, we had to apply rather strict participation criteria. On average, the elderly population, who were recruited in the present study, take more medicines than younger adults, and several medicines can affect sodium balance. To avoid misinterpretations, residents with specific diseases and those who were taking medicines that could significantly affect sodium balance were not included in the present study. Consequently, out of 448 retirement home residents, less than 15% (N = 62) were eligible for participation. Therefore, the sample size of the present study is smaller than in some other studies which investigated salt intake in the general population; it is also smaller than 100 people indicated by the WHO technical meeting report as the likely number for a sufficient estimate of sodium intake in a given population “with a 95% confidence interval about the mean of consumption of +/− 12 mmol/d” [60]. Nevertheless, the method of 24 h urine collection (considered to be a “gold standard”) instead of FFQ was used to estimate sodium and potassium intake in the present study. Additionally, the completeness of urine collection was ascertained through urinary creatinine excretion and urinary volume measurements. We believe both of these resulted in more reliable data and consequently increased the value of the reported results. To our knowledge, our study is at the moment the only one that reported sodium intake among residents of retirement homes. Therefore, no direct comparison of the results with other studies can yet be performed to extend its generalisability. Finally, although participants were asked not to change their dietary habits during the study, it cannot be completely excluded that information provided to participants about high salt intake being a risk factor for noncommunicable chronic diseases in the invitation to the study might have influenced their 24 h sodium and potassium intake. Yet the subjects did not prepare the food by themselves, as it was prepared by professional cooks, therefore this effect should have been minimised.

## 5. Conclusions

The salt intake in residents of retirement homes in the Goriška region, especially in men, exceeds the WHO recommended daily intake of less than 5 g, but is lower than in the Slovene adult population aged 25–65 years. Similar to the population of Slovene adults, the salt intake in retirement home residents is higher in men than in women. The mean daily potassium intake was below the WHO suggestions of at least 3.51 g potassium per day. In our subjects, the Na/K ratio was lower, thus more favourable than in the noninstitutionalised population. We believe that a more favourable Na/K ratio observed in residents of retirement homes, as compared to general, noninstitutionalised population, resulted from the regular daily consumption of freshly prepared food by professional cooks and limited the intake of processed food high in sodium. Because of the rather strict inclusion criteria that had to be applied due to the population studied, and the absence of any other comparable study on a similar population, the generalizability of the reported results will have to be confirmed by future research.

## Figures and Tables

**Table 1:** Anthropometric characteristics and results of 24 h urinary sodium and potassium excretion in residents of retirement homes (*N* = 47).

	Men (*N* = 17)		Women (*N* = 30)		Total (*N* = 47)		Difference between Gend
	Mean	SD	Mean	SD	Mean	SD	*p* Value
**Age (years)**	81.4	10.9	84.3	6.2	83.2	8.2	0.322
**Height (cm)**	166.3	5.7	156.4	5.8	160.0	7.4	<0.001
**Mass (kg)**	79.0	15.0	66.7	13.5	71.1	15.1	0.006
**BMI (kg/m^2^)** ^1^	28.6	5.5	27.3	5.7	27.8	5.6	0.459
**BSA (m^2^)** ^2^	1.87	0.16	1.66	0.16	1.74	0.19	<0.001
**Urinary volume (L/day)**	1.75	0.38	1.37	0.48	1.51	0.48	0.008
**Urinary creatinine (mmol/day)**	9.4	1.8	6.1	1.5	7.3	2.3	<0.001
**Urinary Na excretion (mmol/day**)	155.3	47.8	111.8	34.1	127.6	44.4	<0.001
**Sodium intake (g/day)**	3.6	1.1	2.6	0.8	2.9	1.0	<0.001
**Salt (NaCl) intake (g/day)**	10.1	3.1	7.3	2.2	8.3	2.9	<0.001
**Urinary K excretion (mmol/day)**	60.5	13.6	46.1	17.7	51.3	17.6	0.006
**Potassium intake (g/day)**	2.6	0.6	2.0	0.8	2.2	0.8	0.006
**Ratio Na/K**	1.54	0.46	1.52	0.50	1.53	0.48	0.886

^1^ BMI—body mass index. ^2^ BSA—body surface area according to Du Bois and Du Bois formula (1916) [59].
